# A qualitative study examining young peoples’ perceptions and adherence to COVID-19 public health guidelines in Ireland

**DOI:** 10.1186/s12889-023-16757-7

**Published:** 2023-09-26

**Authors:** Tara M. Breslin, Rose Galvin, Aoife Mare Foran, Orla T. Muldoon

**Affiliations:** 1 School of Medicine, Trinity College Dublin, Dublin 2, Ireland; 2 School of Allied Health, Faculty of Education and Health Sciences, Ageing Research Centre, University of Limerick, Limerick, Ireland; 3 Department of Psychology, Faculty of Education & Health Sciences, University of Limerick, Plassey Park Road, Limerick, Ireland

**Keywords:** COVID-19, SARS-COV-2, Attitudes, Public health guidelines, Adherence, Young people, Qualitative

## Abstract

**Background:**

Public health measures are the main intervention to stop the spread of COVID-19. They rely on the adherence to everyday health behaviors, and depend on those at high and low personal risk of serious disease to comply. Young people are crucial to stemming community transmission, and are often living in shared housing and at a stage of their lives with more economic uncertainty than older groups. Public health messaging has relied on the mantra that we are ‘in it together,’ despite very diverse experiences of the pandemic across different groups. The central aim of this research is to understand and optimize young peoples’ engagement with public health guidelines with the view to improve future adherence with public health initiatives.

**Method:**

Twelve young people were interviewed as part of this research, ranging from 18 to 24 years. Interviewees were chosen to ensure that there was a diverse range of opinions within the participant pool. Interviews were semi-structured with open questions and the flexibility to explore the topics of interest that arose. All interviews were fully transcribed and analyzed using thematic analysis.

**Results:**

This study found that participants deemed the consequences of lockdown a greater threat than infection with SARS-COV-2. Participants expressed concerns about the government’s handling of the pandemic. Some felt young peoples’ interests were not represented by authorities. There were concerns that messaging was inaccurate, difficult to understand, and filled with statistical and medical jargon. These perceptions underpinned a sense that the guidelines could be broken in good conscience as well as result in accidental breaches of the guidelines. Though wider community factors were often cited as having a positive influence on health behavior, differences and division were seen to inspire trust or adherence.

**Conclusion:**

These findings provide an insight into the psychological, financial and physical difficulties young people face as a consequence of pandemic public health measures and lockdowns in particular. They highlight the need for better communication with young people to support and embed trust in authorities and the scientific and political community.

**Supplementary Information:**

The online version contains supplementary material available at 10.1186/s12889-023-16757-7.

The COVID-19 pandemic has thrown up many challenges to public health. At the centre of public health effort in Ireland, and many other countries, was an effort to slow the spread of SARS-COV-2 virus. To this end, public health guidance recommended a range of restrictions on everyday behaviors, that heretofore have not been experienced by many in the Western world [1–5]. For some, the threat of COVID-19 is great and so the restrictions offer personal as well as public health protection [6, 7]. For others, most notably the young and those without pre-existing health conditions, the personal threat of COVID-19 can be perceived as low [5–7]. For this cohort, engagement with public health measures for the greater good to slow the spread of COVID-19 is crucial. In this paper we consider, using an in-depth qualitative approach, how young people make sense of the COVID-19 guidelines, as well as the challenges faced by those seeking to maximize adherence in low-risk populations.

Public health measures have shown to significantly reduce the risk of catching COVID-19 such that they are the main interventions to stop the spread of COVID-19 [3, 5]. Although vaccine roll out has been an important element of the response, particularly in wealthier countries, evidence suggests that vaccines are less efficacious in preventing spread of SARS-COV-2 than previously thought [8–10]. Despite COVID-19 immunizations, transmission of the virus is ongoing. Therefore, the threat of new variants and waning vaccine effectiveness indicates that certain public health measures such as self-isolation when possibly or actually infected, avoidance of crowded locations, masking and hand washing are likely to remain as a main source of prevention for some time [5]. For this reason, understanding and facilitating adherence to public health guidelines amongst all demographic groups remains an important research priority.

Adherence to public health guidelines were the mainstay of the public health response in Ireland in the first year of the COVID-19 pandemic. Overall estimates are that 6,200 people died due to a COVID-19 related death in the first two years of the pandemic [11]. Comparatively speaking, Ireland’s COVID-19 public health response resulted in lower overall mortality per million people when considered in relation to comparable countries such as Scotland and Northern Ireland or those further afield such as the USA or Germany [12]. Public health recommendations were first introduced in Ireland in March 2020, followed by a country-wide lockdown between March and May 2020. In October 2020, a further six-week long lockdown was put in place. After a sharp surge in cases, on December 29^th^, 2020, another country-wide lockdown was established until May 2021. Particularly germane to the cohort in this study, schools and universities were closed and educational offerings were online, unemployment increased from 400,000 to over 1 million people [12] with an associated economic cost of €100 million [11, 12]. Recommendations also included a 2-m social distancing, mandatory face-coverings in all indoor public spaces, travel limited to a 5km radius from one’s home except in exceptional circumstances, and prohibition on social gatherings including weddings, funerals, and graduations. Our participants were interviewed in June 2021.

The recommended health protective behaviors and young people’s willingness to adhere to guidelines have potential costs. Unsurprisingly then, those who have a higher perceived risk of contracting the virus are perhaps more adherent [3, 7, 13, 14]. In a wide range of international studies, perceived personal threat in terms of morbidity or mortality from COVID-19 is a major determinant of adherence [6, 15]. Groups that are not at high risk of adverse outcomes from the disease (i.e., young people; [4, 16, 17]) show lower levels of adherence to guidelines. That said, the majority of young adults report adherence with public health guidelines in quantitative surveys. However, the match between peoples’ self-reported adherence in surveys and their actual behavior is not always strong due to social desirability effects [18, 19]. A qualitative investigation into the engagement with and perceptions of the guidelines therefore offers a more nuanced understanding of young people’s engagement with public health in the context of COVID-19.

However, willingness to engage with the guidelines is only one side of the coin that determines healthy behavior. As a cohort, young people are more likely to have less economic security, reside in insecure or shared housing, and rely on public transport. Thus, the ability to adhere to COVID-19 restrictions is more difficult because of this lack of access to private transport and housing, and insecure employment. Young adulthood also represents a particular developmental period where the pursuit of both career and relationship goals are paramount; not least because they underpin pathways into emerging adulthood. These goals drive young peoples’ social, educational, and health behaviors across early adulthood. Therefore, this developmental stage is associated with the establishment of their social independence but also educational and occupational goals that are the foundational elements of adult life. These developmental imperatives, securing jobs and qualifications, therefore are also perceived as mandatory activities for young people in Ireland and elsewhere. These imperatives were at odds with public health restrictions.

## The current study

A key way in which national and international agencies have sought to engage people with public health advice is by emphasizing solidarity across age, gender and ethnonational groups [12]. Whilst quantitative survey methods offer an overview of adherence, this approach is likely to be affected by demand characteristics and socially desirable responding given widespread awareness in Ireland of the ‘In This Together’ mantra. For this reason, a qualitative study is an important addition to the existing literature**.** Adopting this approach allowed us to consider the impact of young peoples’ engagement with the restrictions in Ireland. Given their lower-than-average personal risk of morbidity and mortality due to COVID-19 infection, as well as their centrality to the transmission, development of new clusters and intergenerational transmission this was an important cohort to consider. Also, because of their developmental stage in life, young people may face challenges that differ from other age cohorts when they try to adhere to COVID-19 restrictions. In short, young people are crucial to efforts to stem ongoing community transmission, despite their comparatively lower personal risk. Given this reality the central aim of this research was to understand young peoples’ engagement with public health guidelines. We argue that this understanding is crucial not just for COVID-19 but for other public health crises that may arise in the future.

## Method

We interviewed young people (6 male; 6 female) as a part of this research, completing 12 interviews once data saturation was reached. Even small sample sizes (N < 10) in qualitative research can reach saturation; the point where little new information is obtained [20]. Participants ranged from 18 to 24 years. All participants were contacted through their higher education institution or by a publicized call to participate on social media. Participants were theoretically sampled to ensure that there was a mix of urban and rural (6 and 6 respectively), and male and female (5 and 7 respectively). One participant identified as a member of an ethnic minority group. Information about the candidates’ age, gender, living situation and employment or education status was also gathered to ensure a broad variety of participants as the study progressed. This was not undertaken to achieve representativeness but rather to ensure that a range of views was represented in the research. Two participants identified themselves as healthcare workers during the COVID-19 pandemic. All participants were currently or formerly enrolled in third level education. Only one participant was no longer in education. Full demographic details of participants are available in table 1 of the appendix.

### Ethical protocol

The study received ethics approval from the Faculty of Education and Health Sciences, University of Limerick, Research Ethics Committee (2021_06_20_EHS). Before the interviews were conducted, the participants were asked to read an information sheet and sign a consent form. This information included how their anonymity would be maintained and how all data would be analyzed and used.

### Interview process

Four higher education institutions and universities were contacted by a member of the research team (University of Limerick, Institute of Technology Sligo, Institute of Technology Carlow, and Trinity College Dublin) and they were asked to circulate the information sheet and contact details of this person. Colleagues of the research team referred candidates for interview. A social media post on Twitter was circulated three times to invite participants to interview. This made clear that the interviews were being conducted by a fellow student. This information was also made clear on the information sheet and consent form in an effort to reduce the power differentials and demand characteristics between the interviewer and participant. Interviews of this type by a peer have been shown to improve the authenticity of participant responses [21].

Participants were contacted by telephone after a time for the interview was mutually agreed. Semi-structured recorded telephone interviews were conducted to avoid person-to-person contact and the risk of virus transmission. This also facilitated interviews of a range of individuals from many areas, both rural and urban, in Ireland. An interview guide provided a loose structure within which to explore the topics of interest, and participants were prompted to expand on relevant and interesting responses. The interviewer used open questions allowing the interviewer to follow the participant’s responses, rather than adhering rigidly to the schedule.

The interview guide was developed with four broad categories of questions: young peoples’ lived experiences since the COVID-19 outbreak; factors linked to adherence to public health guidelines; factors that hinder adherence amongst young people, and young peoples’ views of the vaccine. We aimed for questions that were easy to answer, and would allow participants to talk about themselves, and their past and current lives. The intention was that broad and open-ended questions would facilitate a descriptive narrative that would include talking about any issues participants faced when engaging with public health guidelines.

### Approach to analysis

All interviews were conducted, and audio recorded by an app (Apple IOS “voice memos”). The 12 recordings were transcribed fully. A qualitative approach, specifically a theoretically informed thematic analysis [1] was chosen here to explore how participants experience and live with COVID-19. Our analysis was informed by our theoretical interest in young peoples’ understanding and engagement with public health guidelines and we focused our analysis at both the semantic level and beyond. It is not our intention to provide a sense of the proportions of people reporting enactment of each health behavior. Indeed this is not the aim of qualitative enquiry [13, 14]. Rather, our sampling strategy and data analysis was purposeful. Participants were recruited through universities, higher education and training schemes as well as through social media to ensure we had a range of participants. Our analysis proceeded with line-by-line coding of the transcripts in order to reveal similarities and commonalities in the issues raised by participants. These codes were used to cluster similar concerns and were then used to build potential themes. Calculations of inter-rater reliability have been shown to be unsuited and of little value [22]. Coding is a verifiable and transparent process built on reading and re-reading of transcripts which was carried out by two authors (RG, TB). Subsequently, line level codes were identified and shared between three members of the author team (RG, TB, OM). The next level of analysis involved reviewing the data and codes. In doing so, we considered how they could be amalgamated into overarching elements while maintaining the multiplicity of many of the initial codes into higher level sub-themes. Amalgamation of codes were informed by our research question and agreement between the authors that the overarching themes represented the original codes. Finally outlining the themes, as well as selecting quotes that illustrate these themes, was agreed. Our analysis resulted in three themes which are detailed fully below.

## Results

Three major themes were identified from interviews. These were a) messaging and young peoples’ adherence, b) the social and psychological impact of the restrictions, and c) young peoples’ feelings of social and personal responsibility during the pandemic.

### Theme 1: messaging and young peoples’ adherence

Behaviors associated with lowering the risk of SARS-COV-2 spread can be split into high-cost and low-cost behaviors [6]. A low-cost behavior might be wearing a face covering and frequent handwashing. High-cost behaviors include adhering to self-isolation or social distancing. Adherence with low-cost behaviors was reported as higher than adherence with high-cost behaviors. All participants reported regularly wearing a face covering (face mask) inside, washing hands, and social distancing. By contrast, the high-cost behaviors were associated with lower adherence. Participants explicitly linked poorer adherence to high-cost behaviors to the changing guidelines and contradictory messaging across agencies. Leaks about planned changes to guidelines were also seen as problematic as was inconsistent advice from Irish agencies when compared to other governments’ and current World Health Organisation (WHO) advice. In the below quote the participant linked rule changes to ‘burn out’ and poorer adherence. Participants reported fatigue with the restrictions as being directly related to rules change:

*“Social distancing mask wearing easy peasy no worries on that... [the guidelines] were reasonably well communicated on the news in like TV advertising, that sort of stuff… the problem I had with the government’s response, mainly, was with how often the rules changed… I think that led to a lot quicker rate of burn out from the rules. I think it made people raise their eyebrows unnecessarily to the government’s rules. I think the actual communicating of the rules was fine. I think it was more the constant changing of them was confusing people.” #10*

Many young people struggled to understand the public health announcements. All participants were current or former undergraduate students, and therefore had a high level of education. Many young people did not understand medical jargon or have a scientific background to understand the often-daily press conference held by experts from the Irish NPHET. Our participants believed communicating with the public in a clearer fashion would help increase awareness and understanding, leading to a higher rate of adherence [3]. Another participant suggested that joint press conferences between the government and the emergency team were counterproductive because they appeared to contradict each other. Overall, the sense from participants was that these communications could undermine rather than promote adherence to public health guidelines. Here are two illustrative quotes:

*“A lot of times there’s been conflicting messages between the government and NPHET. I think there should be like probably instead of individual press conferences…just to be joint press conferences or for them to agree on one thing before, you know, like a statement is made…” #12*

*“… everybody, public representative that comes on the radio seems to have a different story and when they’re called upon, ‘oh the last person said this and that,’ they try to revert to what this person said and they all have different varying opinions.” #1*

One participant praised some journalists for breaking down information from the briefings to more understandable, bite-sized pieces of information.

*“Yeah the media has a lot of times has actually been more clear in phrasing things and making things accessible and I don’t understand why that wasn’t the government’s first concern...Things should’ve been done like that from the get go because if you’re trying to get a majority of people to comply with regulations that are a matter of life and death then things should be like as easy to understand as possible.” #6*

Whilst this was seen as positive, the leaking of proposed changes to public health guidelines to the media before a decision or statement was made by NPHET or the government was seen as problematic, potentially confusing and undermining trust. One participant stated:

*“[how do you think they could improve their communication with people?] Not leaking information before you make the announcements would be great. Some actual transparency would be excellent which they lacked for the most part. Because everything, everything, we all found out everything from leaks from the Dáil. The entire time.” #7*

This leaking of information did not inspire confidence in the authorities, reducing trust, and increasing non-adherence.

*“...do I trust them? Like I don’t know do I trust them… I think I do trust them but are they way off the mark? like they’re spouting new stuff every week and I don’t know do they know themselves what they’re saying?” #1*

Participants also pointed out differences in the public health guidelines in Ireland in comparison to other countries. The less severe lockdowns abroad stood in stark contrast to the severe lockdown in Ireland, which led to frustration and a feeling of burnout in many young people. Again, this was reported as reducing adherence.

*“I trust them to get everybody vaccinated. I trust the science… but do I trust the people themselves? Absolutely not. Because they have already messed this up to an embarrassing extent. Ireland had one of the longest lockdowns in Europe and maybe I think the world…their attitude towards it has been absolutely arrogant. So, no I don’t trust them” #7*

Participants did not understand why Ireland’s policies were different to other countries’ policies. This was something that participants needed to understand to feel supportive of the restrictions present in Ireland. In the below quote, the participant is confused by the fact that sporting events in Ireland (hurling often viewed an important Gaelic sport) have such radically different live audience numbers compared to events occurring in the UK and Europe. He states:

*“When I see hurling matches there’s only 200, 300 people allowed into them and look at Wembley, the stadium in the UK, and the Euros [UEFA European Championship football tournament] and there’s 70 000, 60 000 people at the gates. So, there’s something amiss there…” #1*

Overall, young people were actively engaged and thinking about the public health guidelines. They were trying to ensure that they reduced risks. However, unclear, confusing, and frequently changing messaging, as well as discrepancies and contradictory information were articulated as significant causes for lack of confidence in authorities. As a result, trust in the government and public health officials was undermined and adherence with guidelines damaged.

### Theme 2: the social and psychological impact of the restrictions

All participants noted the lockdown experience as the most difficult part of their pandemic experience. Feelings of social detachment, loneliness and loss were routinely reported. There was a perception that young peoples’ needs and wants, different from that of older and younger generations, were not factored into decisions regarding the public health restrictions. Keeping vulnerable groups safe from COVID-19 depends on everyone’s behavior to keep case numbers low. Mostly, participants reported that their adherence to restrictions arose from feelings of obligation toward vulnerable family and friends. For instance, participants stated:

*“It was kind of a stressful time.  To make sure that you weren’t bringing home any virus… so [I] had to be really careful with that… by staying by the guidelines for the most part, it was easier to hope that you wouldn’t bring anything home to your family” #9*

*“Not seeing people was difficult like having to stay away from friends and stuff. But then it was hard in a way but I wouldn’t have felt comfortable in a lot of those situations anyway. Especially when cases were really really high” #6*

As reflected in this latter quote, young people reported being lonely and isolated due to being unable to participate in active social lifestyles resulting in damage to their social circles. On the other hand, they were asked to carry on as normal by employers or during work placements. The following two quotations illustrate how work life was required to proceed in person even though there was no opportunity to develop social contacts.

“Apart from work, no I didn’t... no relationships, no friends… and both my sisters are working in the hospital as well and they haven’t met anyone. And would say [they are] finding it hard to start a relationship now openly…” #9

“We have no work nights out or even work tag rugby team or anything like that … people would’ve looked forward to those nights out and they’re not on anymore. So that’s a big downer for us inside yeah…” #9

The restrictions then rather than the threat of COVID-19, were perceived to drastically change young peoples’ live. It was clear that all the participants interviewed understood that they were in a low-risk group compared to older generations. While initial fear may have aided adherence early in the pandemic, by the time of these interviews in the summer of 2021, there was a clear sense that participants felt that they had been ill-treated. This may explain why frustration, sometimes referred to as lockdown fatigue, grew among all groups, and particularly in young people.

*“The young people who were following the restrictions got the rough end of the deal as well because they were like, ‘oh it’s all the young people’ [breaking the rules] so they probably locked us down more, if that makes sense?” #7*

As indicated in the above quote, some respondents took the view that their needs were not prioritized nor were their priorities considered important. Indeed, some thought they were seen as the problem as suggested by the phrase ‘they locked us down more.’ Changes to restrictions that might have allowed the reopening of cultural attractions, spaces where young people gathered including third level education institutions, were seen as changes that were not important by authorities. This is despite these spaces being much sought after by our respondents. As a result, it was claimed, adherence dwindled.

*“[Do you think most people are following the rules?] I don’t know to be honest. I don’t think they are. But the younger people I suppose don’t have much reason, but I don’t know are older people doing it as much anymore either. Everyone’s vaccinated, the majority are vaccinated. I don’t know if everyone really cares anymore.” #1*

Participants who admitted to seeking mental health support during the COVID-19 crisis were more nervous in general since March 2020, and more adherent to the guidelines. Participants described new feelings of anxiety in social situations:

“I now would be very uncomfortable with people in my personal space whereas I’ve had no problems before going to like arenas and being in concert crowds full of people. Now, if someone gets close to me in the shop, I will sort of give them a dirty look actually *laughs*” #6

“I wouldn’t be fearful of my own mental health but like I would be fearful of others’ … I don’t know if we can go through a whole other lockdown again…” #9

Finally, participants described occasionally breaking the rules to feel better, to visit family and friends, as they felt there was a greater risk to their mental health than their physical health at that time. The below quote illustrates how the social cost of the lockdowns was sometimes perceived as too high by young people resulting in non-adherence to the guidelines.

*“For college…I needed to be in Dublin, but I couldn’t go four months without seeing my family. I just couldn’t do it… I was just going insane. I needed to see people…I knew what I was doing was wrong, but I just couldn’t do it…I limited the times when I was breaking the rules, but I definitely knew what I was doing…as the weeks and months went on, my guard was lowered and lowered. I suppose fatigue with the regulations. Like its been almost a year and a half now…” #10*

Taken together this theme illustrates the impact of the pandemic was felt by young people largely as a result of the restrictions rather than the pandemic itself. The public health guidelines were perceived as having a high social and psychological cost, and at times young people disengaged from the guidelines to manage feelings of distress.

### Theme 3: young peoples’ feelings of social and personal responsibility during the pandemic

All participants acknowledged that while everyone had made sacrifices and struggled with the regulations imposed since the outset of the pandemic, young people have shouldered a disproportionate amount of blame. Some participants said that the government and public bodies, and the media were to blame for encouraging a negative portrayal of young people. Those in positions of power and influence were seen as being responsible for dividing the public and undermining social solidarity by vilifying young people.

*“I think there’s a lot of people who’d be so quick on to Facebook, like a lot of parents or whatever, … They’d be so quick to hop on Facebook, but I think as well, there was a few TDs who were definitely fueling the fire a little bit and who were kind of making comments about young people… they weren’t [all] but there were one or two who weren’t helping.” #11*

Young people, as well as feeling that they were themselves represented in contradictory ways, highlighted the contradictions apparent in the behavior of those in positions of power. Young people pointed to those in authority who defied the guidelines. In the below quote the participant makes reference to this feeling by referring to an event known as ‘Golfgate’ in which many politicians, a judge and an EU commissioner attended a golf tournament and indoor dinner and reception, in breach of one of the lockdowns.

*“…don’t make it like such a condemnation on everyone. Being like ‘oh eh you guys are doing this wrong’ …  I know they did the whole #InThisTogether stuff but … that annoys me because the entire government has not adhered to restrictions like GolfGate and all that. Sure, we’re in this together and then they don’t vaccinate staff at hotels and stuff?” #7*

There was a perceived lack of age and gender, socioeconomic, ethnic, and cultural representation which was seen as problematic. This was viewed as prioritizing the needs of one portion of society, the middle aged and middle-income groups, over younger and less affluent groups. It was also seen as a factor that interfered with people’s willingness to engage with the public health guidelines. One participant mentioned the lack of representation from government and on NPHET.

*“There’s not much representation… they are all older men. There’s not representation from younger people or people from different sectors… if you had information coming from people you trusted and people you relate to they [the guidelines] would make more sense” #4*

As a result, participants were also worried that their concerns were not being considered. Young people had concerns that were both short term and longer term. In the longer term, the economic and social impacts of the restrictions, having witnessed the 2007/’08 economic crisis and noting the ongoing presence of a climate crisis, were often writ large. Participants mentioned their fears for the future and the oncoming instability, and their belief that the government was not doing enough to prevent these consequences.

*“…worried about my own people my age going forward. Who’s going to deal with the consequences of it...with all these PUP (Pandemic Unemployment Payments) our economy is going to suffer like. And there’s probably going to be a crash and prices are gone so high and I feel like it’s all going to come down to what’s happened in the last few years. And I saw a thing about how our generation being the first generation worse off than the previous generation….”  #3*

Some participants showed clear resentment towards the government, public health officials, and the media. One example highlighted multiple times was the decision to reopen restaurants, bars and other indoor public spaces as well as allow household visits during the winter of 2020 resulting in a deadly surge in COVID-19 cases in the community. Some thought the reopening after the 2021 lockdown was too slow, others thought it was too fast. Irrespective of opinion, there was a lack of trust, as well as resentment and frustration with the authorities. These feelings facilitated young peoples’ disengagement from public health policy, and wider government and national policy on all fronts.

Participants felt ignored and that the message of solidarity was merely political rhetoric. For example, one participant highlighted the disparity between the government’s “#InThisTogether” policy and their actions. Some felt there was a lack of leadership from elected individuals, such that the government and opposition were treating public health as a political football. Individuals who felt the situation was politicized and manipulated by authorities seemed to be less likely to engage with guidelines. As a result, some participants thought that governmental decisions had endangered life:

“I trust the public health officials. I don't think that the government has the best interests in mind… so clear to see at Christmas. NPHET told them either open indoor dining or you can do [household] visits but not both. And they did [both]... obviously the government has to make decisions that will affect the economy, but I think this year there's been such a focus on money as opposed to human life and I think that's so sad.” #6

“I think they’re treating it as a bit of a political game as well…… I think they're trying to fend off so many different things and they're not willing to take any risks or make any big decisions. They're really conservative about everything” #3

Some felt that too much power was left in the hands of unelected, public health bodies such as NPHET:

*“I  think your aul Tony Holohan is actually kind of dangerous. Like he’s just so unwilling to accept, do you know like the whole PCR or the antigen test? Like he tweeting something about basically that they don’t work and just what he said wasn't true. And there was people like I follow people on twitter so and they were just like ‘makes no sense because there's science to back all these things up that contradicts everything he says.’” #3*

This lack of trust undermined adherence. Interestingly individuals who admitted to breaking the guidelines reported that others broke the regulations routinely. For instance, in the following quote the participant suggests the ‘whole parish’ broke the regulations to attend a sporting fixture (here referred to as winning the county final), which of course is highly unlikely.

*“I don’t want to be like a rebel or anything. To be honest it like it never really concerned me…we won a county final back in October and the whole place went wild so that was nearly the whole parish. So, if you asked the whole parish what they thought of it, it might’ve been of the same opinion as me judging by their reaction…” #1*

Overall, this theme reflected the sense that young people believed they were asked to sacrifice their social and educational lives, feelings that their concerns were not taken into consideration and that they were blamed for cases when surges happened. None of these perceptions served to bolster young peoples’ solidarity with others in the effort to manage the pandemic or their personal adherence to the restrictions.

## Discussion

There is no doubt that the COVID-19 pandemic has thrown up many challenges to implementation and engagement with public health measures. In this study we examined how young people in Ireland understood and engaged with these measures. Importantly, young people were not always those likely to be direct beneficiaries of the measures and early in the pandemic it became apparent that they were low-risk members of the national group. Their engagement with public health efforts was required to protect health service capacity for those who were vulnerable to the worst effects of COVID-19. What is clear from the interviews is that young people were listening and engaged with issues arising because of infections, but also because of the associated public health restrictions. Our findings suggest that the messaging, the nature of the restrictions and the representations of their own and others’ adherence had a major impact on young peoples’ perceptions and engagement with public health initiatives.

Turning first to public health communications. For the most part, public health initiatives place a strong emphasis on messaging. However, messaging without listening is not communication. Our participants belief that their views and concerns were not represented in the development of restrictions in Ireland, a relatively small country with a negligible democratic deficit. This finding highlights the need for all elements of society to be represented, and to be seen to be represented, if public health advice is to be embraced across a population. All population cohorts need to feel their concerns have been considered and that they are represented within decision-making bodies. Young people who, due to their career and life stage are likely to be perceived as insufficiently expert are at particular risk of being placed outside of the decision-making processes. This can mean important voices are not at the table, making it more likely that young people will not be engaged. Previous research has shown that young people are more trusting of government when there is transparent decision making [23], clear communication and accountability [24]. For this reason, it is also important that public health initiatives move beyond ideas of messaging and think instead of two-way communication.

There appeared to be a clear difference between young peoples’ appreciation and engagement with low-cost behaviors as compared to higher cost or more complex behaviors [6]. The black-and-white nature of low-cost behaviors made them simple to comprehend and follow, as well as less inconvenient by definition. Our findings also highlight the high cost of restrictive public health guidance. Because these restrictions are likely to be particularly hard hitting for young people, they were viewed as troublesome and at times were divisive. In part this is because higher cost guidelines are controversial due to the burden they extract from people and society. However, frequent changes to high-cost rules were perceived to be, and in reality, were likely driven by political concerns in many jurisdictions over the course of the pandemic [20]. These changes and associated indecision were seen as particularly damaging by young people. It undermined trust because it politicized the health advice. This made it easier and more likely for young people to disengage from public health advice.

Added to this, there were contradictions apparent in the advice available from the WHO and the Irish NPHET and public health teams in other nations. These types of contradictions undermine the well-established need for consistency in public health communications [6, 15]. Changes to advice, or contradictory advice delivers ambivalence, undermining adherence. Clear consistent regulations sustain adherence at a higher level [15]. Contradictory communications of this nature are particularly damaging to adherence amongst cohorts who are at limited risk of severe diseases, such as young people. It also points to an urgent need for a clear consistent approach to public health advice by authorities, such as the EU and the WHO. Evident disagreement and confusion between government and public bodies was a source of mistrust. Trust is critical to the public health engagement [25–27]. Trust is earned via listening and responsive communications as well as consistency. Our sense is that the importance of trust in promoting adherence may be particularly influential in other jurisdictions beyond Ireland. Research from Ireland in 2012 noted that of eight participating countries, Ireland had the second highest degree of health literacy [28]. Health literacy is likely to independently support adherence to public health guidelines even where trust in government or government agencies is low as people understand the need to protect themselves. However where health literacy is low, as it is in about 40% of the Irish population, more must be taken on trust for people to engage with the regulations. Actions that change regulations must be explained and justified fully to the public to prevent cynicism and to ensure trust is not squandered. In a global world, regulations that appear illogical or draconian in comparison to other jurisdictions must also be explained clearly and logically. It is incumbent on policy makers to ‘make the policy make sense’ to people in order for trust and adherence to be maintained.

In many regards then our participants suggested their compliance and engagement with the public health guidelines was socially mediated. Participants felt that public opinion was biased in its narrative of who was responsible for spikes and clusters. Our participants had a sense that they were perceived as blameworthy for surges in infections. Equally, participants believed that the authorities were not attuned to the difficulties that they faced and that they were breaching lockdowns. Irrespective of the truth of either narrative, this speaks to an antagonism between generations that undermines the solidarity and cohesion so central to public health initiatives. It also speaks to the need for government and public health agencies to acknowledge and value the trust people place in them. This requires them to be above reproach in their own engagement with public health initiatives and to ensure that any commentary, however inadvertent, does not blame any sector of society for the spread of SARS-COV-2.

### Strengths and limitations

A key strength of the current study was that it interviewed young people and allowed nuances in their understanding to come through. The in-depth qualitative design of this study is a key strength detailing young peoples’ experiences and behaviors. We believe that the young people were candid as they were interviewed by another young person who was an undergraduate on a research studentship at the time of the interviews. For this reason, the overview of adherence is likely to be reliable, unaffected by socially desirable responding, and thus, an important addition to the literature. The current research also had some limitations. All participants interviewed were attending or had attained a third level education qualification. Four participants were current health science students or qualified health care workers, with a fifth student currently studying science. No participants interviewed identified themselves as having an anti-vaccination or conspiracy orientation, and so this relatively small cohort nationally is not represented in the sample. And though there were three non-Irish born participants, there were no participants who did not speak English. A longer recruitment period may have enabled the recruitment of non-English speaker participants, or those not in third level education. Therefore, this study has not investigated the effect of a full or partial language barrier on understanding and adherence with public health guidelines. The knowledge gap between the public health officials’ understanding and young peoples’ understanding of outbreaks, information and guidelines may be higher than is evident in this sample with a comparatively high level of education.

## Conclusion

The COVID-19 pandemic has resulted in many challenges for society in Ireland and globally. In this study we highlight the difficulties that young people in Ireland faced during the first 15 months of the pandemic with a specific aim of understanding their adherence to public health guidelines. Our findings highlight that young people were actively interested and engaged in the public health effort. Lockdowns were experienced as difficult because of their interference with social, educational, and occupational tasks of early adulthood. Because of their high cost, young peoples’ required restrictions to be grounded in logic and evidence. Contradictory positions or changing public health advice were experienced as problematic and bred mistrust. Our findings suggest that the importance of prioritizing trust in support of behavioural adherence and public health decisions cannot be overstated.

### Supplementary Information

**Additional file 1:** **Appendix: Table 1.** Participant Table.

## Data Availability

The datasets generated and analyzed during the current study are not publicly available due to the potential for participants to be identified by reference to their location and age in a country as small as Ireland. Anonymized transcripts are available from the corresponding author on reasonable request.
