# Supplementary material for: A qualitative study examining young peoples’ perceptions and adherence to COVID-19 public health guidelines in Ireland
Source: BMC Public Health. 2023 Sep 26;23:1864. doi: 10.1186/s12889-023-16757-7 (PMC10523624; doi:10.1186/s12889-023-16757-7)
Supplement: Supplementary file 1 — Additional file 1: Appendix: Table 1. Participant Table. [file 12889_2023_16757_MOESM1_ESM.docx]

Appendix: table 1: Participant Table

| Participant | Gender | Rural / urban | Ethnicity | Nationality |
| --- | --- | --- | --- | --- |
| 1 | Male | Rural | White | Irish |
| 2 | Female | Rural | White | Irish |
| 3 | Male | Urban | White | Irish |
| 4 | Female | Urban | White | Irish |
| 5 | Female | Rural | White | Irish |
| 6 | Female | Rural | White | Non-Irish born |
| 7 | Male | Rural | White | Non-Irish born |
| 8 | Male | Urban | Non-White | Non-Irish born |
| 9 | Female | Urban | White | Irish |
| 10 | Male | Rural | White | Irish |
| 11 | Female | Urban | White | Irish |
| 12 | Male | Urban | White | irish |
